# Correlation of a combined serum biomarker panel with the composite physiologic index in connective tissue disease-associated interstitial lung disease

**DOI:** 10.3389/fphys.2026.1768803

**Published:** 2026-02-09

**Authors:** Haiyan Zhou, Jingyun Li, Rui Zhong, Yang Zhang, Yue Zhou, Huipan He, Zhumin Sun

**Affiliations:** Department of Rheumatology, Shuyang Hospital of Traditional Chinese Medicine, Suqian, Jiangsu, China

**Keywords:** composite physiologic index, connective tissue diseases, disease severity, interstitial lung disease, serum biomarkers

## Abstract

**Background:**

To evaluate the diagnostic performance of a combined serum biomarker panel for connective tissue disease-associated interstitial lung disease (CTD-ILD) and its correlation with disease severity.

**Methods:**

This retrospective, cross-sectional study enrolled 200 CTD patients during October 2023 and October 2025, classifying them into CTD-ILD (n = 86) and CTD (n = 114) groups. General clinical characteristics, routine laboratory parameters, serum Krebs von den Lungen-6 (KL-6), surfactant protein A (SP-A), and surfactant protein D (SP-D) levels, serum ferritin (SF), pulmonary function, and the Composite Physiologic Index (CPI) were compared. The predictive power of the biomarker panel was evaluated via receiver operating characteristic (ROC) curves and DeLong’s test, while correlations with the CPI were assessed via Spearman’s correlation analysis.

**Results:**

The groups were comparable in baseline characteristics and routine laboratory parameters. The CTD-ILD group exhibited significantly higher levels of C-reactive protein (CRP), erythrocyte sedimentation rate (ESR), and all four serum biomarkers (KL-6, SP-A, SP-D, and SF) (all *P* < 0.05), alongside impaired pulmonary function and a higher CPI (*P* < 0.05). The combined biomarker panel achieved an area under the curve of 0.980 (95% CI: 0.964–0.996), with 94.20% sensitivity and 95.60% specificity, significantly outperforming individual biomarkers (all *P* < 0.05). All biomarkers demonstrated moderate to high positive correlations with the CPI (*r* = 0.620, 0.520, 0.495, 0.402, respectively; *P* < 0.001).

**Conclusion:**

The combined serum biomarker panel demonstrated strong discrimination for CTD-ILD in this retrospective cohort and was significantly associated with disease severity.

## 1 Introduction

Connective tissue diseases (CTDs) represent a group of systemic disorders characterized by dysregulated autoimmune responses as their core pathological mechanism. This category includes conditions such as systemic sclerosis (SSc), rheumatoid arthritis (RA), polymyositis/dermatomyositis (PM/DM), Sjögren’s syndrome (SS), systemic lupus erythematosus (SLE), and mixed CTD (MCTD) (Yoo et al., 2022; Beydon et al., 2024; Wang et al., 2024; Izuka et al., 2025). Clinically, CTDs affect not only joints, muscles, skin, and blood vessels but also frequently involve internal organs, particularly the lungs (Lewandowska et al., 2025). Epidemiological studies show that approximately 15%–60% of CTD patients develop interstitial lung disease (ILD), with the highest incidence observed in SSc, where it stands as a leading cause of mortality (Drimus et al., 2025; Boyle et al., 2025). Consequently, the early identification and assessment of CTD-associated ILD (CTD-ILD) have become a major focus of shared interest in the fields of rheumatology and pulmonology.

ILD comprises a spectrum of disorders featuring diffuse pathology of the pulmonary interstitium. Fundamental pathological alterations include alveolar epithelial cell injury, inflammatory responses, fibroblast activation, and collagen deposition, ultimately leading to irreversible pulmonary fibrosis and impaired gas exchange/ventilation (Kamiya et al., 2024; Vasarmidi et al., 2025; Nishioka et al., 2024). The pathogenesis of CTD-ILD is closely linked to immune-mediated inflammation, wherein aberrant expression of autoantibodies and inflammatory cytokines plays a significant role (Hirano et al., 2024). However, the clinical presentation of CTD-ILD is often non-specific, with some patients exhibiting mild or even subclinical symptoms in the early stages. Conventional imaging and pulmonary function tests (PFTs) present limitations for early diagnosis. Although high-resolution computed tomography (HRCT) is considered the diagnostic gold standard for ILD, its utility for frequent dynamic monitoring is constrained by cost and radiation exposure concerns (Velazquez Guevara et al., 2023; Johnson et al., 2023). PFTs provide an objective measure of ventilatory and diffusive capacity impairment but lack sensitivity, typically showing abnormalities only after substantial disease progression has occurred. Therefore, there is a pressing need to identify sensitive, specific, and serially measurable serum biomarkers to facilitate the early diagnosis, disease evaluation, and prognosis prediction of CTD-ILD.

In recent years, serum biomarkers have gained prominence in ILD research due to their advantages of convenience, non-invasiveness, and suitability for repeated assessment. Among these, Krebs von den Lungen-6 (KL-6), surfactant protein A (SP-A), surfactant protein D (SP-D), and serum ferritin (SF) have been extensively reported to associate with ILD (Tzang et al., 2025; Conticini et al., 2025; Miadlikowska et al., 2025). KL-6 is a high-molecular-weight mucin-like glycoprotein predominantly secreted by damaged and regenerating type II alveolar epithelial cells. Its elevated serum levels reflect the extent of alveolar-capillary barrier injury. Substantial evidence confirms that KL-6 is not only significantly elevated in idiopathic pulmonary fibrosis (IPF) but also holds diagnostic and prognostic value in CTD-ILD patients (Alvarez et al., 2024; Zhou et al., 2023). SP-A and SP-D, belonging to the pulmonary surfactant protein family, play roles in immune regulation and host defense. Primarily produced by type II alveolar epithelial cells and airway epithelial cells, increased levels of these proteins indicate alveolar epithelial damage and inflammatory activity (He et al., 2025). SF, an acute-phase reactant and key molecule in iron metabolism, is intimately involved in inflammatory and immune processes. Recent studies suggest that elevated SF levels correlate with disease activity, intensity of inflammation, and the degree of fibrosis in various autoimmune disorders (He X. et al., 2024).

Nevertheless, individual serum biomarkers exhibit limitations in the diagnosis and monitoring of CTD-ILD. Combining multiple biomarkers holds promise for enhancing diagnostic sensitivity and specificity, thereby overcoming the constraints of single-marker analysis and creating a more comprehensive and reliable assessment tool. For quantifying ILD severity, the composite physiologic index (CPI), derived from multiple PFT parameters, is widely used and provides a robust reflection of integrated pulmonary impairment, serving as an important prognostic tool. A significant correlation between serum biomarker levels and the CPI would suggest their utility for disease stratification and treatment monitoring, underscoring their clinical relevance.

Against this backdrop, because the CTD-ILD–specific diagnostic value and incremental benefit of combining these serum markers (rather than using them in isolation) remain insufficiently defined in real-world CTD cohorts, this retrospective study analyzed 200 CTD patients to investigate the diagnostic utility of a combined panel of serum KL-6, SP-A, SP-D, and SF for CTD-ILD. Furthermore, it evaluated the correlation between these biomarkers and ILD severity, as measured by the CPI. By comparing the performance of individual markers versus the combined panel, we aim to provide practical evidence to support screening/triage and monitoring of CTD-ILD and to inform future multicenter prospective validation.

## 2 Materials and methods

### 2.1 Sample size estimation

The primary outcome of this study was the diagnostic efficacy of combined serum biomarkers for CTD-ILD. According to a previous report (Xu et al., 2025), the area under the receiver operating characteristic (ROC) curve (AUC) for a single serum biomarker in detecting ILD is approximately 0.80, whereas we expected the AUC of the combined detection to increase to 0.90. With a two-sided significance level of α = 0.05 and a power (1-β) of 0.80, the sample size was estimated using PASS version 15.0. The calculation indicated that at least 74 patients were required per group. Considering an approximate 10% dropout or incomplete data rate, the final target sample size was set at no fewer than 85 patients per group. Consequently, this study ultimately included 86 patients in the CTD-ILD group and 114 in the CTD group, which met the requirements for statistical analysis.

### 2.2 Patient population

A retrospective analysis was conducted by screening all hospitalized patients diagnosed with CTDs at Shuyang County Traditional Chinese Medicine Hospital between October 2023 and October 2025 using the electronic medical record system. Eligible patients were included if they met the pre-specified inclusion and exclusion criteria, with no additional sampling or subjective selection. The final study cohort comprised 200 patients. The cohort comprised 27 cases (13.50%) of SSc (van den Hoogen et al., 2013), 68 cases (34.00%) of RA (Aletaha et al., 2010), 32 cases (16.00%) of PM/DM (Lundberg et al., 2017), 52 cases (26.00%) of SS (Aringer et al., 2019), 13 cases (6.50%) of SLE (Aringer et al., 2019), 5 cases (2.50%) of MCTD (Alarcon-Segovia and Cardiel, 1989), and 3 cases (1.50%) of undifferentiated CTD (UCTD) (Mosca et al., 1999). All diagnoses conformed to their respective internationally recognized classification criteria. Based on the presence or absence of comorbid ILD, patients were stratified into two groups: a CTD-ILD group (n = 86) and a CTD group (n = 114). The diagnosis of ILD was established according to the Diagnosis and Treatment Guidelines for CTD-ILD issued by the Chinese Rheumatology Association (CRA) (Zou et al., 2022).

Inclusion criteria were as follows: 1) age ≥18 years; 2) fulfillment of the aforementioned diagnostic criteria for CTD and, where applicable, CTD-ILD; 3) no history of immunomodulatory therapy within the 3 months prior to the index date that could potentially influence serum biomarker levels, such as high-dose glucocorticoid pulse therapy, biological agents, cyclophosphamide, mycophenolate mofetil, or rituximab; 4) availability of complete clinical data, including detailed medical records, laboratory results, and imaging findings, sufficient for accurate assessment and group assignment; 5) absence of a history of malignant tumors; and 6) ethics committee approval.

Exclusion criteria encompassed: 1) coexistence of other established causes known to induce ILD, including occupational dust exposure, drug-induced lung injury, radiation pneumonitis, or hypersensitivity pneumonitis; 2) severe heart failure [New York Heart Association (NYHA) class III-IV] resulting in pulmonary edema or related radiographic abnormalities; 3) active pulmonary infections, tuberculosis, lung neoplasms, or pulmonary embolism; 4) other major severe systemic comorbidities, such as significant hepatic or renal dysfunction, or hematological malignancies; 5) pregnancy or lactation; and 6) presence of psychiatric disorders or cognitive impairment.

### 2.3 Collection of general clinical characteristics

General clinical data for all enrolled patients were collected and documented through a comprehensive review of the hospital’s electronic medical record system. The information obtained included sex (male/female), age (in years, calculated at the time of hospital admission), and smoking history (defined as having smoked a cumulative total of ≥100 cigarettes). The duration of CTD (in months) was calculated as the interval from the date of initial diagnosis to the date of the current hospitalization. Furthermore, the specific type of CTD was recorded for each patient. Based on the applicable international classification criteria, patients were categorized into the following diagnostic groups: SSc, RA, PM/DM, SS, and SLE, with the number and percentage of cases for each type being documented.

### 2.4 Conventional laboratory investigations

Fasting venous blood samples were obtained from the antecubital vein of all patients on the morning following hospital admission. A fully automated hematology analyzer (Mindray, model BC-5100) was employed to determine the white blood cell count (WBC), red blood cell count (RBC), platelet count (PLT), and hemoglobin (HGB) concentration. The C-reactive protein (CRP) level was measured using an immunoturbidimetric assay performed on a fully automated biochemistry immunoanalyzer (Roche Diagnostics, model Cobas c 702) with the manufacturer’s original reagents. The erythrocyte sedimentation rate (ESR) was assessed using a fully automated dynamic ESR analyzer (Pulisen Fully automatic Erythrocyte Sedimentation Rate Analyzer: XC-20B).

### 2.5 Serum biomarker assays

Fasting venous blood samples, collected concurrently with those for routine tests, were allowed to clot at room temperature and subsequently centrifuged at 3,500 rpm for 20 min. The resulting supernatant serum was aliquoted and stored at −80 °C in an ultra-low temperature freezer (Panasonic, Japan, model MDF-U3386S) until analysis. Prior to assay, serum samples were thawed and equilibrated to room temperature. In addition to standard clinical evaluation (including HRCT and pulmonary function testing), serum KL-6, SP-A, SP-D, and SF were measured because these biomarkers are biologically linked to alveolar epithelial injury and have been reported in prior studies to be associated with the presence and disease burden of ILD, including CTD-ILD (He et al., 2021; Takahashi et al., 2000; Gono et al., 2010). Saliva liquefied sugar chain antigen (KL-6) was detected using the KL-6 assay kit from Hunan Yonghe Sunshine Biotechnology Co., LTD. The magnetic particle chemiluminescence method was adopted, and the detection was strictly carried out in accordance with the instructions. SP-A and SP-D levels were measured using human enzyme-linked immunosorbent assay (ELISA) kits (Cusabio Technology LLC, Wuhan, China) according to the manufacturer’s instructions. Absorbance was read at 450 nm using a full-wavelength microplate reader (Thermo Fisher Scientific, model Multiskan MK3) for concentration calculation. Ferritin (SF) was detected by electrochemiluminescence using the fully automatic biochemical immunoassay analyzer (model: Cobas c 602) produced by Roche Diagnostics and the original matching reagents. All assays were conducted by dedicated laboratory personnel who were blinded to the clinical groupings of the subjects.

### 2.6 Pulmonary function testing

PFTs were performed for all patients during periods of clinical stability using the MasterScreen PFT System (Jaeger, Germany; model MasterScreen) by the same experienced technician. To ensure measurement accuracy, the instrument underwent daily volume calibration with a standard 3.0-L calibration syringe provided by the manufacturer, in addition to gas calibration using a gas mixture containing 0.3% carbon monoxide, 10% helium, 21% oxygen, and balance nitrogen. The measured parameters included total lung capacity (TLC) determined by body plethysmography, forced vital capacity (FVC) obtained from the average of the three best tests after maximal inspiration followed by a forced and rapid expiration to residual volume, and forced expiratory volume in one second (FEV1) recorded simultaneously. The diffusing capacity for carbon monoxide (DLCO) was examined using the single-breath method. All pulmonary function measurements were conducted in strict adherence to the Chinese Expert Consensus on Standardized Pulmonary Function Testing in Adults (Zhu, 2022).

### 2.7 Assessment of ILD severity

The severity of ILD was assessed using the CPI, calculated based on the established formula: CPI = 91 - [0.65 × percent predicted DLCO (DLCO%pred)] - [0.53 × percent predicted FVC (FVC%pred)] + [0.34 × percent predicted FEV1 (FEV1%pred)], where higher scores indicate more severe disease (Wells et al., 2003).

### 2.8 Statistical analysis

Statistical analyses were performed using SPSS software (version 27.0). The normality of continuous variables was assessed with the Shapiro-Wilk test. Data with a non-normal distribution were presented as median with interquartile range [M (P25, P75)], and comparisons between groups were conducted using the Mann-Whitney U test. Normally distributed data were expressed as mean ± standard deviation ( $\bar{x}$ ± *s*), and the homogeneity of variances was verified by Levene’s test. For comparisons between groups with equal variances, the independent samples *t*-test was applied; if variances were unequal, the Welch *t*-test was used. Categorical variables were summarized as frequencies and percentages [n (%)] and were compared using the chi-square (*χ* ^2^) test or Fisher’s exact test, as appropriate. The predictive performance of the serum biomarkers, both individually and in combination, for identifying CTD-ILD was evaluated by constructing ROC curves, with comparisons of AUCs performed using the DeLong’s test. The optimal cutoff value was determined by maximizing Youden’s index (calculated as sensitivity + specificity - 1). The correlations between serum biomarker levels and ILD severity, as quantified by the CPI, were analyzed using Spearman’s correlation coefficient. A significance level of α = 0.05 was adopted for all tests, with *P*-values less than 0.05 considered statistically significant.

Internal validation: To address potential overfitting, the combined biomarker model was internally validated using bootstrap resampling and repeated k-fold cross-validation. For bootstrap validation, 1,000 bootstrap samples were drawn with replacement. In each resample, the model was refit and the optimism (AUC_bootstrap-AUC_original) was estimated; the optimism-corrected AUC was calculated as AUC_apparent minus the mean optimism. In addition, repeated 10-fold stratified cross-validation was performed (100 repeats with different random splits), and the mean cross-validated AUC with standard deviation was reported.

## 3 Results

### 3.1 Comparison of baseline clinical characteristics

A comparison of the baseline clinical characteristics between the CTD-ILD and CTD groups revealed no statistically significant differences in sex, age, smoking history, CTD duration, or distribution of CTD subtypes (all *P* > 0.05) (Table 1).

**TABLE 1:** Comparison of baseline clinical characteristics between CTD-ILD and CTD groups.

Characteristic	CTD-ILD (n = 86)	CTD (n = 114)	*χ* ^2^ */t/Z*	*P*
Male/Female (n)	17/69	24/90	0.050	0.824
Age (years)	58.79 ± 11.27	57.33 ± 11.46	0.897	0.371
Smoking history [n (%)]	12 (13.95)	15 (13.16)	0.027	0.871
CTD duration (years)	5.94 ± 2.57	5.72 ± 2.44	0.615	0.539
CTD type [n (%)]			11.113	0.070
SSc	14 (16.28)	13 (11.40)	—	—
RA	32 (37.21)	36 (31.58)	—	—
PM/DM	16 (18.60)	16 (14.04)	—	—
SS	17 (19.77)	35 (29.82)	—	—
SLE	2 (2.33)	11 (9.65)	—	—
MCTD	4 (4.65)	1 (0.88)	—	—
UCTD	1 (1.16)	2 (1.75)	—	—

CTD, connective tissue disease; ILD, interstitial lung disease; SSc, Systemic sclerosis; RA, rheumatoid arthritis; PM/DM, Polymyositis/Dermatomyositis; SS, Sjögren’s syndrome; SLE, systemic lupus erythematosus; MCTD, mixed connective tissue disease; UCTD, undifferentiated connective tissue disease.

### 3.2 Comparison of routine laboratory parameters

No statistically significant differences were found between the CTD-ILD and CTD groups in the serum levels of WBC, RBC, PLT, or HGB (all *P* > 0.05). In contrast, serum CRP and ESR levels were significantly elevated in the CTD-ILD group compared to the CTD group (*P* < 0.05), as presented in Table 2.

**TABLE 2:** Comparison of routine laboratory parameters between CTD-ILD and CTD groups.

Parameter	CTD-ILD (n = 86)	CTD (n = 114)	*t/Z*	*P*
WBC (×10^9^/L)	7.45 ± 2.06	7.33 ± 1.99	0.399	0.690
RBC (×10^12^/L)	4.29 ± 0.55	4.35 ± 0.53	0.864	0.389
PLT (×10^9^/L)	240.00 ± 64.18	254.60 ± 59.78	1.656	0.099
HGB (g/L)	126.14 ± 16.86	129.10 ± 14.90	1.313	0.191
CRP (mg/L)	17.10 (11.10, 23.50)	9.70 (8.50, 13.70)	6.061	<0.001
ESR (mm/h)	35.77 ± 15.94	28.00 ± 11.32	3.847	<0.001

CTD, connective tissue disease; ILD, interstitial lung disease; WBC, white blood cell count; RBC, red blood cell count; PLT, platelet count; HGB, hemoglobin; CRP, C-reactive protein; ESR, erythrocyte sedimentation rate.

### 3.3 Comparison of serum biomarker levels

The serum levels of all four biomarkers, KL-6, SP-A, SP-D, and SF, were significantly elevated in the CTD-ILD group compared with those in the CTD group (*P* < 0.05), as detailed in Table 3.

**TABLE 3:** Comparison of serum biomarker levels between CTD-ILD and CTD groups.

Biomarker	CTD-ILD (n = 86)	CTD (n = 114)	*Z/t*	*P*
KL-6 (U/mL)	1117.05 (800.20, 1599.40)	521.55 (408.80, 636.50)	9.228	<0.001
SP-A (ng/mL)	87.56 ± 35.27	49.40 ± 17.47	9.216	<0.001
SP-D (ng/mL)	158.76 ± 62.65	92.40 ± 31.36	9.008	<0.001
SF (ng/mL)	238.65 (169.20, 314.60)	154.95 (112.00, 199.70)	6.251	<0.001

CTD, connective tissue disease; ILD, interstitial lung disease; KL-6, Krebs von den Lungen-6; SP-A, Surfactant protein A; SP-D, Surfactant protein D; SF, serum ferritin.

### 3.4 Comparison of pulmonary function and CPI

The CTD-ILD group exhibited significantly lower values for all measured pulmonary function parameters, TLC, FVC, FEV1, and DLCO, compared to those in the CTD group (all *P* < 0.05). Furthermore, the CPI was significantly higher in the CTD-ILD group, indicating greater disease severity (*P* < 0.05), as summarized in Table 4.

**TABLE 4:** Comparison of pulmonary function parameters and CPI between CTD-ILD and CTD groups.

Parameter	CTD-ILD (n = 86)	CTD (n = 114)	*t/Z*	*P*
TLC (%pred)	67.76 ± 10.80	85.53 ± 8.29	12.695	<0.001
FVC (%pred)	64.45 (56.10, 73.30)	88.65 (81.80, 95.30)	10.685	<0.001
FEV1 (%pred)	68.30 (60.40, 76.50)	89.60 (82.70, 96.10)	10.263	<0.001
DLCO (%pred)	55.30 (46.80, 64.20)	82.80 (74.10, 89.20)	11.026	<0.001
CPI	43.02 (36.71, 52.49)	20.12 (15.99, 26.64)	11.211	<0.001

CTD, connective tissue disease; ILD, interstitial lung disease; TLC, total lung capacity; FVC, forced vital capacity; FEV1, Forced expiratory volume in 1 s; DLCO, diffusing capacity for carbon monoxide; CPI, composite physiologic index.

### 3.5 Predictive efficacy of combined serum biomarkers for CTD-ILD

**FIGURE 1:**
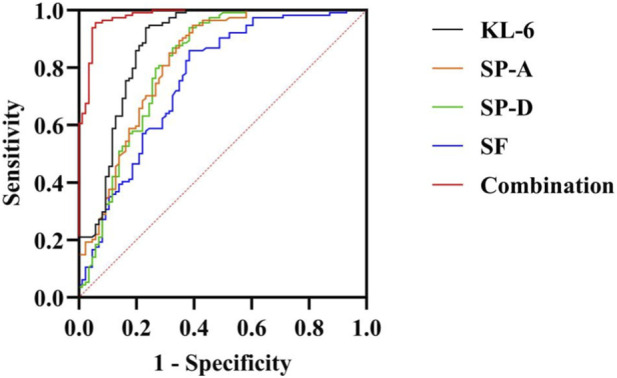
ROC curves for combined and individual serum biomarkers in predicting CTD-ILD. Note: ROC, Receiver operating characteristic; CTD, Connective tissue disease; ILD, Interstitial lung disease.

ROC curve analysis and the DeLong’s test demonstrated that the combined detection of serum biomarkers yielded an AUC of 0.980 [95% confidence interval (CI): 0.964–0.996)], with a sensitivity of 94.20% and a specificity of 95.60%. This predictive performance was significantly superior to that of any individual biomarker assessed alone: KL-6 (AUC = 0.881), SP-A (AUC = 0.823), SP-D (AUC = 0.816), or SF (AUC = 0.758) (Z = 4.116, 5.259, 5.179, 6.312, respectively; all *P* < 0.05). Internal validation results: The combined biomarker model showed an apparent AUC of 0.982. After bootstrap validation (1,000 resamples), the optimism-corrected AUC was 0.978. In repeated 10-fold cross-validation (100 repeats), the mean AUC was 0.975 ± 0.001, suggesting that the excellent discrimination was largely retained after internal validation. The detailed results are presented in Tables 5, 6, and Figure 1.

**TABLE 5:** Predictive efficacy of individual and combined serum biomarkers for CTD-ILD.

Biomarker	AUC (95% CI)	Youden’s index	Sensitivity	Specificity	*P*
KL-6	0.881 (0.829–0.934)	0.706	76.70%	93.90%	<0.001
SP-A	0.823 (0.762–0.884)	0.552	60.50%	94.70%	<0.001
SP-D	0.816 (0.751–0.880)	0.555	61.60%	93.90%	<0.001
SF	0.758 (0.689–0.828)	0.476	61.60%	86.00%	<0.001
Combination	0.980 (0.964–0.996)	0.898	94.20%	95.60%	<0.001

CTD, connective tissue disease; ILD, interstitial lung disease; AUC, area under the curve; CI, confidence interval; KL-6, Krebs von den Lungen-6; SP-A, Surfactant protein A; SP-D, Surfactant protein D; SF, serum ferritin.

**TABLE 6:** Pairwise comparison of AUCs.

Comparison	*Z*	*p*	*AUC* difference	95% CI
Combination-KL-6	4.116	<0.001	0.099	0.052–0.146
Combination-SP-A	5.259	<0.001	0.157	0.099–0.216
Combination-SP-D	5.179	<0.001	0.165	0.102–0.227
Combination-SF	6.312	<0.001	0.222	0.153–0.291

AUC, area under the curve; CI, confidence interval; KL-6, Krebs von den Lungen-6; SP-A, Surfactant protein A; SP-D, Surfactant protein D; SF, serum ferritin.

### 3.6 Diagnostic performance of the combined serum biomarker panel across CTD subtypes

Subgroup analyses stratified by CTD subtype showed that the combined serum biomarker model maintained good discrimination across major subtypes. The AUCs were 0.956 for SSc, 0.978 for RA, 1.000 for PM/DM, and 0.980 for SS, with bootstrap 95% CIs shown in Table 7; rare subtypes (SLE/MCTD/UCTD) were combined as ‘Other CTDs’ to improve the stability of estimation.

**TABLE 7:** Diagnostic performance of the combined serum biomarker panel across CTD subtypes.

CTD subtype	Total (n)	CTD-ILD (n)	CTD (n)	AUC (bootstrap 95% CI)
SSc	27	14	13	0.956 (0.844–1.000)
RA	68	32	36	0.978 (0.946–0.998)
PM/DM	32	16	16	1.000 (1.000–1.000)
SS	52	17	35	0.980 (0.933–1.000)
Other CTDs (SLE/MCTD/UCTD)	21	7	14	1.000 (1.000–1.000)

CTD, connective tissue disease; ILD, interstitial lung disease; SSc, Systemic sclerosis; RA, rheumatoid arthritis; PM/DM, Polymyositis/Dermatomyositis; SS, Sjögren’s syndrome; SLE, systemic lupus erythematosus; MCTD, mixed connective tissue disease; UCTD, Undifferentiated connective tissue disease. Estimates for ‘Other CTDs’ should be interpreted cautiously due to small sample size.

### 3.7 Correlation between serum biomarkers and ILD severity

**FIGURE 2:**
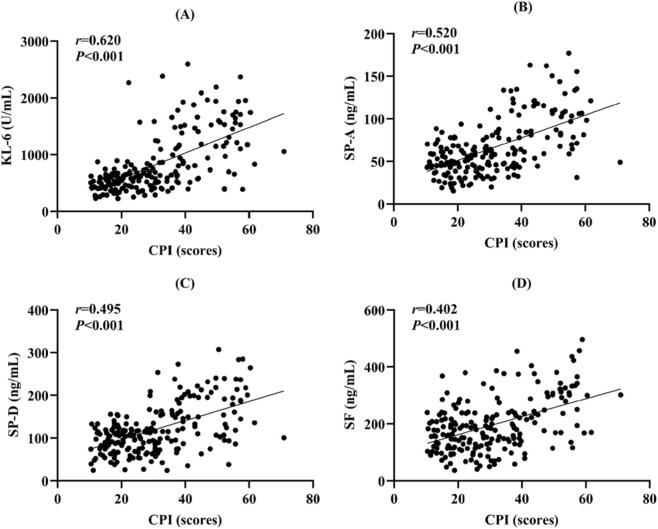
Scatter plots illustrating the correlations between serum biomarker levels and the CPI. Note: **(A)** KL-6 vs. CPI, **(B)** SP-A vs. CPI, **(C)** SP-D vs. CPI, **(D)** SF vs. CPI. CPI, Composite physiologic index; KL-6, Krebs von den Lungen-6; SP-A, Surfactant protein A; SP-D, Surfactant protein D; SF, Serum ferritin.

Spearman correlation analysis revealed that serum levels of all four biomarkers, KL-6, SP-A, SP-D, and SF, demonstrated moderate to high positive correlations with the CPI (*r* = 0.620, 0.520, 0.495, and 0.402, respectively; all *P* < 0.001), as illustrated in Figure 2.

## 4 Discussion

As one of the most common and serious pulmonary complications in patients with CTDs, CTD-associated ILD poses a significant threat to both quality of life and overall prognosis (Bautista-Sanchez and Khanna, 2024; Farquhar et al., 2023). While numerous previous studies have focused on the roles of HRCT and PFTs in the diagnosis and assessment of ILD (Wu et al., 2021; Rahaghi et al., 2023; He W. et al., 2024)these modalities present limitations for repeated/serial assessment and dynamic monitoring. The present study evaluates a more convenient, non-invasive, and highly reproducible detection method through the combined application of serum biomarkers, which may offer a new perspective for clinical diagnosis. Compared to a single biomarker, a panel of multiple markers may reflect pathophysiological changes from different angles, thereby potentially enhancing diagnostic sensitivity and specificity. This combined serological approach may serve as an effective auxiliary tool, particularly in patients with minimal or nonspecific respiratory symptoms in whom ILD may be under-recognized, to help prioritize HRCT evaluation.

Our results demonstrated that serum levels of KL-6, SP-A, SP-D, and SF were all significantly elevated in CTD-ILD patients compared to those with CTD alone. Furthermore, these biomarkers showed moderate to high positive correlations with the CPI, indicating a potential association with the extent of interstitial lung damage and disease severity. ROC curve analysis indicated that the combination of all four biomarkers achieved an AUC of 0.980, with a sensitivity of 94.20% and a specificity of 95.60%, performance which was significantly superior to any individual marker. This finding supports the potential value of a multi-marker panel in the diagnosis of CTD-ILD.

KL-6, the most extensively studied biomarker in ILD, has been previously validated for its diagnostic and prognostic value in CTD-ILD. For instance, studies have reported correlations between KL-6 levels and HRCT scores as well as the degree of pulmonary function impairment (Shin et al., 2024). Our results are consistent with these reports, showing significantly elevated KL-6 in the CTD-ILD group and a strong correlation with the CPI. SP-A and SP-D are also established markers of alveolar epithelial injury, with prior research indicating their elevated levels in ILD patients and association with disease activity. Our study confirmed this and further revealed that while their correlation coefficients with the CPI were lower than that of KL-6, they may still provide clinically relevant information. SF, an acute-phase reactant, has recently been implicated in the inflammatory response and fibrotic progression of autoimmune diseases (Ouyang et al., 2022; Jiang et al., 2022; Maus et al., 2023). The significant elevation of SF in CTD-ILD patients and its positive correlation with the CPI observed in our study suggest that SF may reflect, at least in part, the combined processes of pulmonary inflammation and fibrosis. Notably, these biomarkers are not entirely disease-specific. Prior studies have reported that KL-6 and surfactant proteins (SP-A/SP-D), and in some settings ferritin, may also rise during acute lung injury or intercurrent infections (e.g., pneumonia, acute exacerbations, and viral pneumonitis) (Delgado et al., 2015; Awano et al., 2020; Zhou et al., 2020). Thus, their elevation may reflect both chronic epithelial injury in CTD-ILD and transient spikes related to acute episodes along the disease course. Because our study used single-timepoint sampling, we were unable to distinguish chronic baseline elevation from acute-phase increases; future longitudinal studies with serial measurements (stable phase-acute episode-recovery), combined with infection markers and imaging/physiology, are needed to clarify biomarker kinetics and optimize clinical interpretation.

Mechanistic rationale for the multi-marker panel. From a pathobiological perspective, the four-marker panel may capture complementary but interconnected axes of CTD-ILD: epithelial injury/regeneration (KL-6/MUC1), surfactant system disruption and innate immune dysregulation at the alveolar interface (SP-A/SP-D), and systemic/macrophage-driven inflammation with iron-metabolism perturbation (SF). Experimental evidence suggests that KL-6/MUC1 is not only a marker of injured type II alveolar epithelial cells but may also exert pro-fibrotic effects by promoting fibroblast chemotaxis and supporting fibroblast survival/proliferation (Hirasawa et al., 1997; Ohshimo et al., 2005). SP-A and SP-D, as collectins, modulate macrophage and dendritic cell responses, regulate cytokine production, and help shape the balance between host defense and excessive inflammation in the injured lung (Pastva et al., 2007). In parallel, ferritin can reflect heightened macrophage activation and inflammatory burden in autoimmune hyperinflammatory states, which is relevant to inflammatory-fibrotic progression in CTD-ILD (Ruscitti et al., 2018) and has been linked to ILD activity in specific CTD contexts such as anti-MDA5 dermatomyositis (Gono et al., 2011). Taken together, integrating these markers may better represent the “epithelial injury-immune activation-fibroproliferation” continuum than any single marker alone, which may partly explain the improved discrimination observed with the combined model.

ROC analysis revealed AUCs of 0.881 for KL-6 alone, 0.823 for SP-A, 0.816 for SP-D, and 0.758 for SF. The combination of all four biomarkers significantly increased the AUC to 0.980. This combined approach not only integrates the strengths of each individual marker but also may help compensate for their respective limitations. For example, while KL-6 exhibits high sensitivity, it can also be elevated in some inflammatory lung conditions other than CTD-ILD. SP-A and SP-D may be more sensitive in the early stages of alveolar damage but suffer from lower specificity. SF levels are considerably influenced by the systemic inflammatory state. The panel may improve specificity while maintaining high sensitivity, thereby potentially supporting early diagnosis and stratification. Importantly, subgroup analyses by CTD subtype demonstrated consistently good discrimination across the major subtypes (SSc, RA, PM/DM, and SS), while the pooled “Other CTDs” subgroup should be interpreted cautiously due to the limited sample size.

This study suggests that the combined detection of serum KL-6, SP-A, SP-D, and SF may serve as a potential adjunctive tool for the early screening of ILD in CTD patients, particularly in patients with minimal or nonspecific respiratory symptoms in whom ILD may be under-recognized, to help prioritize HRCT evaluation. Furthermore, the significant correlation of these biomarkers with the CPI indicates their potential not only for diagnosis but also for gauging disease severity, which may inform the development of individualized treatment strategies. For instance, patients with persistently elevated serum markers may be considered for closer follow-up with HRCT or PFTs, with treatment decisions guided by overall clinical assessment. Additionally, these serological parameters could be explored as endpoints in clinical trials, providing objective evidence for verifying the efficacy of new drugs and interventions.

This study has several limitations. Its single-center, retrospective, cross-sectional study, and the limited sample size introduces the potential for selection bias. Although internal validation (bootstrap resampling and repeated cross-validation) was performed to mitigate concerns about overfitting, external validation in independent, multi-center cohorts is still warranted to confirm generalizability and reproducibility. CTD is heterogeneous; although discrimination was generally consistent across major subtypes, rarer subtypes required pooling as “Other CTDs,” and subtype-specific estimates should be interpreted cautiously and confirmed in larger cohorts. Moreover, assay availability and laboratory standardization may differ across institutions, which could affect generalizability; external validation across diverse settings is warranted. The analysis was restricted to four biomarkers (KL-6, SP-A, SP-D, SF) and did not include other potentially relevant markers such as CCL18, MMP-7, or YKL-40, possibly underestimating the comprehensiveness of serological testing. The cross-sectional nature of the study provides data from a single timepoint, precluding observation of dynamic changes in biomarker levels over the course of the disease, and limiting our ability to distinguish chronic baseline elevation from transient increases related to intercurrent infections or acute lung injury/exacerbations. In addition, symptom burden was not uniformly quantified in this retrospective dataset, precluding stratified analyses in patients with minimal or nonspecific respiratory symptoms. Longitudinal monitoring would be more informative for assessing progression and prognosis. Although efforts were made to exclude comorbidities, serum levels can still be influenced by factors such as infection, medication use, and hepatic or renal function, making it difficult to completely avoid confounding bias. Another limitation is that a healthy control group was not available in this retrospective cohort; therefore, baseline reference levels of these biomarkers could not be established. Finally, this study is primarily an observational clinical analysis; the molecular mechanisms underlying the elevation of these serum markers were not explored and require further elucidation through basic science research.

## 5 Conclusion

In this single-center retrospective cohort, serum KL-6, SP-A, SP-D, and SF were higher in patients with CTD-ILD than in those with CTD without ILD. A combined biomarker model provided strong discrimination for CTD-ILD and retained performance after internal validation. HRCT remains the accepted reference standard for definitive CTD-ILD diagnosis; therefore, this biomarker panel should be positioned as an adjunctive screening/triage tool to support risk stratification and to prioritize HRCT evaluation, rather than as a substitute for HRCT. External validation in independent multicenter cohorts is required before clinical implementation.

## Data Availability

The original contributions presented in the study are included in the article/supplementary material, further inquiries can be directed to the corresponding authors.
